# Common Femoral Vein Wall Thickness as a Diagnostic Marker for Behçet's Disease: A Case‐Control Study

**DOI:** 10.1002/hsr2.71296

**Published:** 2025-09-26

**Authors:** Raghad Tarcha, Muttaz Hanna, Mohamad Ali Nahas, Maysoun Kudsi

**Affiliations:** ^1^ Department of Rheumatology, Faculty of Medicine Damascus University Damascus Syria; ^2^ Department of Obstetrics and Gynecology Nürnberg Germany; ^3^ Division of Vascular and Endovascular Surgery, Faculty of Medicine, National University Hospital Damascus University Damascus Syria; ^4^ Faculty of Medicine Damascus University Damascus Syria

**Keywords:** Behçet's disease, diagnostic imaging, oral ulcers, ultrasonography, vein wall thickness, venous Doppler

## Abstract

**Background and Aims:**

Recent publications have highlighted the potential diagnostic value of measuring common femoral vein (CFV) wall thickness in Behçet's Disease (BD). The diagnosis of BD remains a clinical challenge. The objective of this study is to evaluate the utility of venous Doppler ultrasound measurement of CFV wall thickness as a diagnostic tool for BD and to differentiate it from healthy controls (HCs).

**Methods:**

A total of 96 individuals were included: 48 patients with BD and 48 HCs. CFV wall thickness was measured bilaterally using venous Doppler ultrasound. Comparative statistical analyses were performed between groups, and diagnostic cutoff values were determined.

**Results:**

Both right and left whole‐wall thickness (WWT) of the CFV were significantly higher in the BD group compared to HCs (*p* < 0.001). A diagnostic cutoff value of ≥ 0.668 mm for CFV WWT effectively distinguished BD from HCs, with a sensitivity of 89.6% and specificity of 100%. Among BD patients, those with vascular involvement had notably thicker CFV walls. No significant correlations were observed with ocular, neurological, articular, or gastrointestinal involvement, nor with laboratory markers or most treatment modalities.

**Conclusion:**

Ultrasound assessment of CFV wall thickness is a promising, noninvasive diagnostic tool that may aid in differentiating BD from healthy individuals. Its significant association with vascular involvement further supports its role as a potential marker of disease severity.

**Abbreviations**
BD: Behçet disease
BSAS: Behçet's Syndrome Activity Score
CBC: complete blood count
CFV: common femoral vein
CFVWT: common femoral vein wall thickness
CRP: C‐reactive protein
ESR: erythrocyte sedimentation rate
HCs: healthy controls
ICBD: International Criteria for Behcet's Disease
VWT: vein wall thickness
US: ultrasound

## 1 Introduction

Behçet's disease (BD) is a systemic inflammatory condition of unknown etiology, frequently seen in the Silk Road countries, including Syria [1]. The disease usually appears in the third decade of life, with a male predominance [2]. In male patients, the disease is more severe [3], and involvement of major organs is more frequent, resulting in increased mortality due to severe neurological damage, paralysis, and/or severe cognitive impairment [4, 5]. BD progresses in the form of attacks and remissions. Attacks are more severe and recurrent during the initial years of BD, but they become more benign as time passes [5, 6]. Recurrent oral ulcers, genital ulcers, and uveitis are the most common clinical findings in BD, in addition to involvement of other organs, including the vascular system [4, 5, 6]. Vascular involvement in BD is observed in approximately one‐third of patients, with primary involvement of the venous system [7].

Vascular manifestations in this disease are the result of an inflammatory response, with thromboinflammation, endothelial dysfunction, platelet activation, and thrombogenesis directly sustained by neutrophil hyperactivation [8]. Vascular abnormalities in BD are diagnosed using clinical assessments and imaging methods, which include Doppler ultrasound (US), computed tomography, and magnetic resonance angiography [6, 7, 8, 9, 10, 11]. Several recent studies have focused on venous wall thickness in BD, notably Erturk and colleagues, Sevik and colleagues, and Kahveci and Cevval, supporting common femoral vein wall thickness (CFVWT) as a practical diagnostic marker [9, 10, 11]. The first controlled Doppler US study by Alibaz‐Oner et al. [7] showed that vein wall thickness (VWT) increased in the lower extremity veins of BD patients, independently of vascular involvement. Based on several previous data, it has been suggested that measurement of the common femoral vein (CFV) thickness can be valuable, noninvasive, and practical for diagnosing BD [12, 13, 14, 15, 16]. In our report, we aimed to investigate the VWT of the CFV measured using Doppler US in patients with BD and differentiating them from controls.

## 2 Materials and Methods

### 2.1 Study Design, Setting, and Ethics

All data, including sociodemographic and clinical information, smoking status, disease duration, medications, and laboratory results, were collected from BD patients followed at Al‐Muwassat University Hospital and National University Hospital, Rheumatology Department, between September 2023 and September 2024.

Patients who met the inclusion criteria were enrolled. Doppler US was performed on 48 BD patients and 48 healthy controls (HCs) on the day they visited the outpatient clinic or rheumatology department. Patients with evidence of venous insufficiency or inconsistency (> 0.1 mm) between bilateral whole‐wall thickness (WWT) measurements were excluded. HCs were hospital employees or patients with other complaints but no personal or family history of immune diseases. Doppler measurements were performed free of charge after obtaining informed consent.

The sample size was calculated based on the method described by Kadam and Bhalerao [17], resulting in (*n* = 48/group) calculated for effect size 0.6, *α* = 0.05, power 0.8.

Ethical approval for this case‐control study was obtained from the Ethical Committee of Damascus University, Syria (27/8/2023, Approval No: 3440). The study adhered to the Declaration of Helsinki (1975), revised in 2013.

### 2.2 Participants

The study population consisted of two groups admitted to the Rheumatology Department at Damascus University Hospitals: 48 BD patients and 48 HCs. BD diagnosis was established according to the International Criteria for Behçet's Disease (ICBD) [18]. The HC group were hospital staff with no personal/family immune disease and matched by age and sex.

Inclusion criteria: BD patients diagnosed by ICBD criteria [18].

Exclusion criteria: age < 18 years; obesity (BMI > 30 kg/m^2^); pregnancy or breastfeeding; current or past history of thrombosis; chronic venous insufficiency; systemic or immune diseases; malignancy; heart, lung, hematologic diseases; hypertension; diabetes mellitus; cerebrovascular, psychiatric diseases; and infection. Additionally, participants showing > 0.1 mm difference between right and left CFV WWT measurements were excluded to avoid measurement inconsistency due to potential technical or pathological factors; two patients were excluded based on this criterion.

### 2.3 Venous Doppler Ultrasonography

All Doppler US examinations were performed on the same day as clinical assessments by a single expert vascular surgeon with 20 years of experience, blinded to the patients' clinical status. Bilateral CFV, was examined craniocaudally using a high‐resolution Doppler US system (Esaote MyLab system (Esaote, Genoa, Italy), equipped with a high‐frequency linear transducer (10–18 MHz).

The hip was externally rotated, and the knee slightly flexed to optimize visualization and reduce muscle tension. Deep and superficial veins were examined for chronic thrombotic changes, venous insufficiency, recanalization, reflux, and collateral development. Venous insufficiency was assessed in supine and prone positions using the Valsalva maneuver at the saphenofemoral junction and popliteal veins.

VWT of the CFV was measured 2 cm distal to the saphenofemoral junction in the supine position, focusing on the posterior wall to avoid reverberation artifacts. Anterior wall measurements were avoided for the same reason. Two measurements per vessel were taken, and the average was recorded.

WWT of the CFV were measured on the same day by two different radiologists to assess interobserver reliability in ten patients. The measurements were repeated a few days later by the same radiologist to assess intraobserver reliability. Both inter‐ and intraobserver concordances were good for the mean right and left CFV WWT [intraclass correlation coefficient (ICC): 0.881 (95% CI: 0.712, 0.953), *p* < 0.001; ICC: 0.913 (95% CI: 0.812, 0.988), *p* < 0.001, respectively]. Categorizing WWT based on the cutoff value showed perfect agreement (*κ* = 1).

### 2.4 Statistical Analysis

All analyses were performed using the Statistical Package for the Social Sciences software, Version 26 (SPSS Inc., Chicago, IL, USA). Results are presented as mean ± standard deviation, number, and percentage.

Pearson's *χ* ^2^ test was used for categorical variables. Since the data were not normally distributed, nonparametric tests were used for all analyses. Independent samples were compared using appropriate significance tests, such as the Mann–Whitney *U* test and the Kruskal–Wallis *H* test. A two‐sided *p* < 0.05 was considered statistically significant.

A multivariable logistic regression was performed to assess whether CFVWT independently predicted BD diagnosis, adjusting for age, sex, and smoking status.

Receiver operating characteristic (ROC) curve analysis was conducted to evaluate the diagnostic performance of CFVWT. The optimal cutoff was determined by Youden's *J* statistic.

Raghad Tarcha (corresponding author) had full access to data and takes responsibility for the accuracy of analysis.

## 3 Results

A total of 96 participants were included in the present study. The HC and BD groups were comparable in terms of age and sex. Detailed demographic data are presented in Tables 1, 2, 3.

**Table 1:** Demographics and smoking status.

Variable	Controls (*n* = 48)	BD Patients (*n* = 48)	*p*
Age (mean ± SD)	0.582 ± 0.04	0.834 ± 0.15	0.60
Sex (M/F)	30/18	30/18	1.00
BMI	24.1 ± 2.8	25.3 ± 2.6	0.12
Nonsmoker	23 (47.9%)	20 (41.7%)	0.11
Current smoker	18 (37.5%)	26 (54.2%)	0.21
Former smoker	7 (14.6%)	2 (4.2%)	0.09

**Table 2:** CFV wall thickness measurements.

Group	Mean ± SD (mm)	Min–Max (mm)
Controls	0.582 ± 0.04	0.50–0.66
BD	0.834 ± 0.15	0.56–1.16

*Note:* ROC curve analysis yielded an area under the curve (AUC) of 0.95. The optimal cutoff value of CFVWT was 0.668 mm, with sensitivity 89.6% and specificity 100.

**Table 3:** Characteristics of BD patients.

Variable	Category	Number (*n*)	Percentage (%)
Age of patients (years)	< 1	9	18.8
	1–5	22	45.8
	6–10	9	18.8
	> 10	8	16.7
	Total	48	100
Disease activity (BSAS score)	Mean ± SD	16.58 ± 17.38	—
	Range	0–70	—
Clinical Manifestations	Ocular	36	75.0
	Articular	23	47.9
	Vascular	16	33.3
	Neurological	6	12.5
	Gastrointestinal	1	2.1
ESR (mm/h)	Mean ± SD	29.52 ± 22.89	—
	Range	2–115	—
CRP (mg/L)	Mean ± SD	11.26 ± 13.88	—
	Range	1–59	—
Hemoglobin (g/dL)	Mean ± SD	12.67 ± 1.69	—
	Range	9.3–16.7	—
Anemia	Present	24	50.0
	Absent	24	50.0
Colchicine treatment	Yes	15	31.3
	No	33	68.8
Nonbiologic immunosuppressant	Yes	40	83.3
	No	8	16.7
Biologic medications	Yes	10	20.8
	No	38	79.2
Current daily dose of steroid (mg/day)	None	15	31.3
	< 7.5	6	12.5
	7.5–20	11	22.9
	> 20	16	33.3
	Total	48	100

### 3.1 Multivariable Analysis

In the multivariable logistic regression analysis, CFVWT was the sole independent predictor of BD diagnosis (*β* = 27.18, SE = 5.12, *p* < 0.00001), whereas age, sex, and smoking status were not significant.

The mean wall thickness of the common femoral vein (CFVWT) in patients with BD was significantly greater (0.846 mm) than that in controls (0.583 mm) (*p* = 0.000), indicating a statistically significant association between increased CFVWT and BD.

When assessing vascular involvement among BD patients, those with vascular lesions had significantly higher CFVWT (1.01 mm) compared to those without (0.76 mm) (*p* = 0.000), suggesting a robust relationship between vascular involvement and venous wall thickening.

Additionally, BD patients without vascular involvement (*n* = 32) showed significantly higher CFVWT (mean ± SD = 0.75 ± 0.6 mm) compared to HC (*n* = 48, 0.582 ± 0.04 mm), *p* = 0.03. This finding suggests that venous wall thickening may also be present in BD patients without overt vascular disease.

No statistically significant differences were found in CFVWT based on ocular involvement (*p* = 0.766), neurological involvement (*p* = 0.296), articular involvement (*p* = 0.718), or gastrointestinal involvement (*p* = 0.772), indicating a lack of correlation between these organ manifestations and CFVWT.

Regarding disease duration, the highest CFVWT was observed in patients with disease duration > 10 years (0.96 mm), yet the Kruskal–Wallis test showed no significant difference among duration groups (*p* = 0.131).

No correlation was found between CFVWT and disease activity (BSAS) (Spearman's *ρ* = 0.099, *p* = 0.502), ESR (*ρ* = 0.063, *p* = 0.671), or CRP (*ρ* = 0.143, *p* = 0.331), indicating that venous wall thickening is independent of systemic inflammatory markers.

As for anemia, patients with anemia had a lower CFVWT (0.82 mm) than those without (0.87 mm), but this difference was not statistically significant (*p* = 0.247).

Regarding treatment, no significant differences in CFVWT were found between patients treated with colchicine (*p* = 0.929), nonbiological immunosuppressants (*p* = 0.179), or biologic agents (*p* = 0.656).

Steroid dosage (current daily dose) showed a nonsignificant trend toward higher CFVWT with increasing doses. The highest mean thickness was observed in the 7.5–20 mg group (0.93 mm), followed by > 20 mg (0.86 mm), with the lowest values in patients not receiving steroids (0.80 mm) and those receiving < 7.5 mg (0.78 mm) (*p* = 0.059) (Table 4).

**Table 4:** Group comparisons and correlation analyses of common femoral vein wall thickness (CFVWT) in Behçet's disease patients.

Clinical/laboratory feature	*N*	CFVWT (Mean ± SD, min–max)	*p*/correlation coefficient	Test used
Vascular involvement	16	1.01 ± 0.08 (0.9–1.16)	< 0.001	Mann–Whitney *U*
Genital ulcers (yes)	23	0.81 ± 0.14 (0.65–1.07)	0.13	Mann–Whitney *U*
Genital ulcers (no)	25	0.82 ± 0.16 (0.56–1.16)		
Ocular involvement (yes)	36	0.87 ± 0.18 (0.66–1.16)	0.48	Mann–Whitney *U*
Disease duration			*r* = 0.03, *p* = 0.82	Spearman
CRP			*r* = 0.26, *p* = 0.07	Spearman
ESR			*r* = 0.16, *p* = 0.29	Spearman
Current daily steroid dose			*r* = −0.02, *p* = 0.86	Spearman

## 4 Discussion

BD is a chronic, multisystemic inflammatory disorder characterized by recurrent oral and genital ulcers, uveitis, skin lesions, and vascular involvement. Vascular pathology, affecting both arteries and veins, is one of the critical causes of morbidity and mortality in BD patients due to the development of thrombosis, aneurysms, and vascular wall inflammation [19, 20]. The inflammatory process leads to endothelial dysfunction, vascular remodeling, and hypercoagulability, contributing to vein wall thickening and thrombosis [13].

Our study specifically focused on measuring the CFVWT by Doppler ultrasonography in BD patients, comparing them to HC. The rationale behind this approach is the clinical challenge of diagnosing BD especially at early disease stages or incomplete BD presentations. We included BD patients with organ involvement to test marker sensitivity in severe cases and noted as limitation generalizability to mucocutaneous‐only cases.

Our findings demonstrate a statistically significant increase in CFVWT in BD patients compared to HC, consistent with prior studies showing that subclinical vascular inflammation can be detected by noninvasive US methods even in the absence of overt vascular events [12, 13]. This supports the hypothesis that vascular wall thickening is an early and sensitive marker of vascular involvement in BD.

Histopathological studies have shown perivascular infiltration by neutrophils, lymphocytes, and monocytes in BD patients, with associated endothelial cell activation and damage leading to vein wall edema and fibrosis [21]. These microscopic changes correspond well with the macroscopic US findings of increased wall thickness, highlighting the value of US as a diagnostic adjunct.

Interestingly, our study did not find significant correlations between CFVWT and systemic inflammatory markers such as erythrocyte sedimentation rate (ESR) and C‐reactive protein (CRP), nor with clinical disease activity indices like the Behçet's Syndrome Activity Score (BSAS). This indicates that CFVWT may reflect a more chronic and localized vascular remodeling process rather than acute systemic inflammation. These findings align with those of Alibaz‐Oner and colleagues, who also noted that VWT did not directly correlate with systemic inflammatory markers, suggesting a dissociation between ongoing vascular remodeling and systemic inflammatory activity [12].

No significant associations were observed between CFVWT and other BD clinical manifestations, including ocular, neurological, joint, or gastrointestinal involvement. This suggests that femoral vein wall thickening might represent a specific vascular pathology feature independent of other systemic manifestations. Given the heterogeneity of BD, this finding supports the notion that vascular involvement should be assessed and monitored independently to tailor therapeutic approaches appropriately [20].

Although patients with obesity (BMI > 30 kg/m^2^) were excluded, we reported the BMI values of participants in the results to enhance transparency. Given the known relationship between body mass and vascular remodeling, future studies with larger samples may further explore the influence of BMI on venous wall thickness in BD.

Furthermore, our finding that BD patients without vascular involvement had significantly higher CFVWT than HCs supports the hypothesis that subclinical venous inflammation or remodeling may be a common feature of BD, even in the absence of clinically evident vascular events. This highlights the potential of CFVWT as a diagnostic marker not limited to vascular BD subtypes.

Regarding treatment effects, we observed no statistically significant difference in CFVWT among patients receiving colchicine, conventional immunosuppressants, or biological agents, although there was a nonsignificant trend toward increased CFVWT with higher corticosteroid doses. This may be explained by the chronic nature of vascular remodeling, including fibrosis and smooth muscle proliferation, which might not be reversible with immunosuppressive therapy alone [20]. Additionally, corticosteroids' impact on vascular structure can be complex, sometimes promoting fibrosis or impairing repair mechanisms [22].

Our findings align with Alibaz‐Oner et al. [11] and extend to a Syrian cohort. Moreover, systematic reviews [23] and recent work on large‐vessel wall thickness [24, 25] corroborate the diagnostic value of venous wall measurements in BD. Furthermore, Kırmızıer et al. [26] reported increased pulmonary artery wall thickness in BD patients, while Ağaçkıran et al. [27] described similar changes in the inferior vena cava. These findings, along with those of Kutluğ Ağaçkıran et al. [28] who linked vascular wall thickening to disease severity, support the broader concept of systemic vascular remodeling in BD. Our results regarding CFVWT are consistent with this pattern and reinforce the role of venous ultrasonography as a diagnostic and potentially prognostic tool.

### 4.1 Limitations

This study is limited by its two centers design and relatively small sample size, which may affect the generalizability of the results. In addition, the cross‐sectional nature of the study precludes assessment of longitudinal changes in venous wall thickness or response to treatment. The absence of histopathological confirmation of vascular changes is another limitation.

## 5 Conclusion

This study demonstrates that the measurement of the CFVWT can serve as a valuable, noninvasive diagnostic tool to differentiate BD from patients and healthy people. The significantly increased CFVWT in BD patients, particularly those with vascular involvement, supports the hypothesis of subclinical vascular inflammation as a hallmark of BD. While no significant associations were found between CFVWT and other clinical manifestations or inflammatory markers such as ESR and CRP, the clear distinction between BD and HC groups emphasizes the diagnostic utility of CFVWT.

## Author Contributions

**Raghad Tarcha:** writing – original draft, data curation. **Muttaz Hanna:** writing – original draft, conceptualization. **Mohamad Ali Nahas:** writing – review and editing. **Maysoun Kudsi:** writing – review and editing. All authors have read and approved the final version of the manuscript.

## Ethics Statement

This study was approved by the Ethics Committee of Damascus University (27/8/2023, Approval No.: 3440).

## Consent

Written informed consent was obtained from all participants.

## Conflicts of Interest

The authors declare no conflicts of interest.

## Transparency Statement

The lead author Raghad Tarcha affirms that this manuscript is an honest, accurate, and transparent account of the study being reported; that no important aspects of the study have been omitted; and that any discrepancies from the study as planned (and, if relevant, registered) have been explained.

## Data Availability

The data sets used and/or analyzed during the current study are available from the corresponding author on reasonable request. The corresponding author, Raghad Tarcha, had full access to all data in the study and takes complete responsibility for the integrity of the data and the accuracy of the data analysis.
